# A new perspective on binaural beats: Investigating the effects of spatially moving sounds on human mental states

**DOI:** 10.1371/journal.pone.0306427

**Published:** 2024-07-31

**Authors:** Salomé Sudre, Richard Kronland-Martinet, Laetitia Petit, Jocelyn Rozé, Sølvi Ystad, Mitsuko Aramaki

**Affiliations:** 1 CNRS, PRISM (Perception, Representations, Image, Sound, Music), Aix Marseille Univ, Aix-en-Provence, France; 2 LPCPP (Laboratoire de Psychologie Clinique, de Psychopathologie et de Psychanalyse) (EA 3278), Aix Marseille Univ, Aix-en-Provence, France; La Sapienza University of Rome, ITALY

## Abstract

When individuals are exposed to two pure tones with close frequencies presented separately in each ear, they perceive a third sound known as binaural beats (BB), characterized by a frequency equal to the difference between the two tones. Previous research has suggested that BB may influence brain activity, potentially benefiting attention and relaxation. In this study, we hypothesized that the impact of BB on cognition and EEG is linked to the spatial characteristics of the sound. Participants listened to various types of spatially moving sounds (BB, panning and alternate beeps) at 6Hz and 40Hz frequencies. EEG measurements were conducted throughout the auditory stimulation, and participants completed questionnaires on relaxation, affect, and a sustained attention task. The results indicated that binaural, panning sounds and alternate beeps had a more pronounced effect on electrical brain activity than the control condition. Additionally, an improvement in relaxation was observed with these sounds at both 6Hz and 40Hz. Overall, these findings support our hypothesis that the impact of auditory stimulation lies in the spatial attributes rather than the sensation of beating itself.

## Introduction

Electrical activity in the human brain is generally classified in 5 types of brain waves that are characterized by their frequency range: Delta (0.5–4 Hz), Theta (4–8 Hz), Alpha (8–12 Hz), Beta (12–30 Hz) and Gamma (>30 Hz). The activity in each of these frequency bands has been correlated with a particular cognitive state: Delta frequencies are generally associated with deep sleep, Theta frequencies with light sleep and meditation state, Alpha frequencies with a state of relaxation, Beta and Gamma frequencies with a state of active consciousness [1]. Different studies have shown that these brain waves can be stimulated and even synchronized with stimulations in different modalities (visual and auditory [2]). Concerning the auditory modality, some findings suggest that auditory stimuli could be used to modify the neural activity of the brain [3–5]. Among such stimuli, we can mention binaural beats (BB) which were introduced in 1839 by Heinrich Wilhelm Dove. This phenomenon is observed when two pure tones of close frequencies, each in one ear, are presented to the listener. The resulting sound is perceived as a third sound which frequency corresponds to the mean frequency value of the two initial pure tones and with an amplitude modulation relative to the difference between the two frequencies. Experiments conducted on anesthetized cats revealed that these perceived beats are thought to originate in the superior olivary complex of the brainstem, which is the first structure in the ascending auditory pathway to receive information from both ears [6, 7].

Many researchers have been interested in BB and their effect on brain waves. Nevertheless, the reliability of their effects remains contradictory. This can be explained by the stimuli and protocols used, which differ from one study to the other (for a review, see [8]). Furthermore, the way in which auditory stimuli are constructed or generated is often not reported in studies, or with little rigor. The results are then difficult to reproduce. For example, Karino et al. [9] showed that the brain responses evoked by Theta frequency BB contained a specific spectral component which frequency corresponded to that of the BB. Kasprzak [10] observed a decrease in spectral density in the Alpha and Beta frequency ranges and an increase in Theta during Alpha frequency BB presentation. Pratt et al. [11] observed that the event-related potential (ERP) component P50 amplitude was higher with BB at 3 Hz than 6 Hz. The reverse was shown for N100 and P200. These three ERPs components are generally associated with the perceptual processing of the physical characteristics of the stimulus. The following year, Pratt et al. [12] replicated their experiment by adding an experimental condition: monaural beats. Monaural beats are similar to binaural beats, except that the two pure tones are presented simultaneously in both ears. The resulting beat originates at the cochlear level. All sounds elicited a P50, N100, and P200; however, this study neither showed that BB have a different nor greater effect than monaural beats, nor that the cortical distribution of evoked potentials is altered by these stimuli. Schwarz and Taylor [13] found a visible 40 Hz oscillation during BB listening. However, this oscillation was weaker than with monaural beats. This study therefore showed that, although less pronounced than monaural beats, BB may influence the brain activity. López-Caballero and Escera [14], on the other hand, failed to replicate these results. Gao et al. [15] recorded the electrical brain activity of participants while listening to Delta, Theta, Alpha and Beta frequency BB in pink noise. The results showed that for Delta frequency BB, the relative power decreased in Theta and Alpha frequency bands while it increased in the Beta frequency band. The same was observed for Alpha frequency BB. For Beta frequency BB, only a decrease in Theta was shown. Finally, for Theta frequency BB, no significant change in relative power was observed, regardless of the frequency range tested. The authors also calculated a phase-locking value (i.e., an index to quantify the synchronization of the brain electrical signals at specific frequencies). The authors concluded that BB could induce changes in brain connectivity. In particular, the results revealed functional connectivities between anterior and posterior areas in the Theta frequency band with Delta, Alpha and Theta frequency BB. In a recent study, Seifi Ala et al. [16] failed to confirm such changes in functional connectivity but showed an increase in relative power in the Theta frequency band only with Theta frequency BB.

In addition to these studies, BB have been studied for their potential cognitive and therapeutic benefits such as improving mood and state of alertness and attention or reducing anxiety (for a review, see [17–19]). Padmanabhan et al. [20] showed a decrease in anxiety score for the group that listened to BB compared to a control group. Weiland et al. [21] obtained the same result with Alpha frequency BB. However, the authors also demonstrated the same reduction in anxiety with musical and environmental sounds. Gantt et al. [22], on the other hand, were interested in the use of BB in military personnel with chronic stress complaints following deployment. For that purpose, they measured the heart rate variability. Results showed a decrease in low frequency heart rate for the BB group (theta frequency) and an increase for the control group after the intervention. The reverse was shown for high frequency heart rate. In addition, participants who listened to BB reported being less stressed than those in a control group. Others have measured the effect of BB in the frequency ranges corresponding to Beta, Theta, and Delta waves during a vigilance task from objective and subjective measures on 29 participants [23]. They reported more targets and fewer false alarms with BB corresponding to the Beta wave frequencies. Reedijk et al. [24] used an attentional blink (AB) task, in which participants are presented with a series of stimuli containing two target stimuli. While participants generally detect the first target quite easily, they fail to detect the second one when presented quickly after the first one. This AB phenomenon could be attributed to a misallocation of attentional resources between the two targets. The authors reported that Gamma frequency BB (40 Hz) dramatically reduced the AB effect in participants with low spontaneous eye blink rate (thought to represent a low level of striatal dopamine). This result was not observed with Alpha-frequency BB (10 Hz). In a similar vein, Colzato et al. [25] showed that Gamma frequency BB (40 Hz) lead to a decrease in the global precedence effect and thus, an increase in visual attentional focus, compared to a control tone in a global-local task. On the contrary, other studies reported no effect on attention or vigilance during stimulation with beats at 4 Hz (Theta), 16 Hz (Beta) or 40 Hz (Gamma) [26–28]. In conclusion, while many studies have investigated BB, the disparity in results and methodologies employed does not provide a consensus on their effect at cortical oscillation or behavioral levels.

### Binaural beats and spatial hearing

Interestingly, the BB phenomenon can be considered, from an acoustical point of view, as equivalent to a spatial displacement of a virtual sound source around the listener. In fact, we can show that BB can be identically reproduced with temporal phase shifts of the same signal presented in one of the two ears.

The phenomenon of BB appears when two pure tones of close frequencies *f*_1_ and *f*_2_ are presented simultaneously and separately in each ear. The signals are noted *s*_*l*_(*t*) = *sin* (*2πf*_1_*t*) and *s*_*r*_(*t*) = *sin* (*2πf*_2_*t*) for the left and right ears respectively. The resulting sound is perceived as a third sound that can be defined as a sinusoidal signal of carrier frequency $\frac{f_{1}+f_{2}}{2}$ (i.e., the mean frequency) and of modulating frequency $\frac{f_{1}-f_{2}}{2}$ (i.e., half of the difference between the two frequencies). The perception of the BB is optimal around 440 Hz for a carrier frequency lower than 1500 Hz and for a modulating frequency lower than 35–40 Hz [29, 30]. Above these values, two distinct sounds are perceived.

On the other hand, the human hearing system uses several perceptual cues to locate a sound source in a 3D space [31]. In the horizontal plane, the main cues result from the differences in the signals reaching the two ears (binaural cues), i.e. interaural time differences (ITD, phase shifts) and interaural level differences (ILD, sound level difference). When ITD and/or ILD vary over time, the source is perceived as moving in space around the listener. ITDs are predominant for frequencies below 1000 Hz, and ILDs are predominant for signals with frequencies above 1500 Hz. In addition to ITD and ILD, the spectral cues related to the filtering by the body of the listener, mainly the head, the pinna and the auditory canal, are important for sound localization, particularly in the vertical plane. They are characterized by so-called head-related transfer functions (HRTF), for left and right ears. These functions are specific to each individual and are determined by the morphology of the listener.

Based on these considerations, we can make the link between the phenomenon of BB and a spatial displacement of a sound source based on ITD cues since it can easily be shown that the two signals simply differ by a linear phase shift in both the ITD and the BB cases. Considering the BB signals *s*_*l*_(*t*) and *s*_*r*_(*t*), the signal presented to one of the ears, e.g. the signal *s*_*r*_(*t*), can be rewritten as:

$$\;s_{r}\left(t\right)=sin\;\left(2\pi f_{2}t\right)\;=sin\;\left(2\pi f_{1}t\;+\Phi \left(t\right)\right)\;\;\;\text{with}\;\;\;\;\Phi \left(t\right)=2\pi (f_{2}-f_{1})t$$ (1)

This means that a linear phase shift *Φ*(*t*) is the only difference between *s*_*r*_ (*t*) and *s*_*l*_(*t*). In other terms, this is equivalent to presenting a same signal of frequency f_1_ at both ears and adding a linear phase *Φ*(*t*) to one of the 2 ears. This is equivalent to the ITD based on a phase shift induced by a source displacement around the listener. For a frequency difference *f*_2_−*f*_1_ of less than 3 Hz, it was shown that the sound is perceived as moving to the right and left sides of the head [30]. As presented previously, the BB are better perceived with a carrier frequency that would not exceed 1500 Hz. This can be explained by the fact that the ITDs are predominant for frequencies below 1000 Hz.

Finally, the BB should not be confused with monaural beats (MB) for which the beats can be perceived independently of the frequency range. In monaural conditions, the resulting sound, which is presented to both ears, can be written as:

$$s(t)=sin\;(2\pi f_{1}t)\;+sin\;(2\pi f_{2}t)=2\;sin\;(2\pi \frac{f_{1}+f_{2}}{2}t)\;cos\;(2\pi \frac{f_{1}-f_{2}}{2}t)$$ (2)

In this case, the perception of monaural beats is not due to interaural differences, but to the frequency selectivity of the ear that is determined by the notion of critical bands. Hence, when two frequency components are present within the same critical band, they cannot be distinguished and are perceived as beats or roughness depending on their frequency difference [32].

Our primary objective of our study was to investigate whether the impact of BBs on brain activity, relaxation, and attention could be linked to the spatial attributes of these auditory stimuli. For this purpose, we designed 4 types of stimuli: 1) Monaural beats as a control condition; 2) Binaural beats that are equivalent to simulating a moving source by phase shifting, i.e., based on ITDs simulation; 3) Panning beats that are equivalent to simulating a moving source by intensity modulation, i.e., based on ILDs simulation; 4) Alternate beeps, i.e. short beeps that are presented alternately in the two ears, and that correspond to the discrete version of the BB condition. For each type of stimuli, we considered 2 beating frequencies (6 and 40 Hz) to examine different brain wave frequency ranges. The stimuli were submitted to two groups of participants, one group for each beating frequency value (G6 and G40 groups).

Firstly, given the lack of consensus on this matter in the literature, we aimed to testing the effects of BBs on brain activity and cognition. Specifically, we hypothesized that 6Hz binaural beats enhance relaxation whereas 40Hz binaural beats enhance sustained attention. Consequently, we expected the group exposed to 40Hz binaural beats (G40) to demonstrate improved performance on a sustained attention task. We anticipated an improvement of the score on a relaxation scale in the group exposed to 6Hz BB (G6). In terms of EEG measurements, we predicted that the spectral power in the frequency band corresponding to the stimulation frequency (6 or 40Hz) surpass those of other frequency bands. Secondly, to address our main objective, we assumed that the actual effects of the auditory stimulation on the cognitive state are contained in its spatial attribute rather than the sensation of beating itself and that these effects are larger for stimuli evoking perceptible moving sound sources. Consequently, our expectation was that the observed effects on EEG, sustained attention, and relaxation would be more pronounced in the panning beats and alternate beeps conditions than in the BB condition, and that the effects are larger in all the latter three conditions than in the control condition (monaural beats).

## Material and methods

### Participants

An a priori power analysis was conducted using G*Power version 3.1.9.7 [33] for sample size estimation, using the effect size found by Garcia-Argibay et al. ([17], g = 0.45). The minimum sample size needed with this effect size is N = 16 per group. Forty-two volunteers were initially recruited for this experiment both from the Aix-Marseille University community and from the general public between February and April 2023. These volunteers were recruited from a broader audience who responded to calls for participation broadcast during science outreach events aimed at the general public. To participate, they had to meet the following criteria: no neurological nor psychiatric history, no specific medication, normal hearing and normal or corrected vision. Data from 4 participants were removed from the study due to missing electrophysiological or behavioral data (2 females) and excessive artifact on the EEG (1 female, 1 male). Thus, a total of 38 participants (17 females, 21 males), seven of which are left-handed (4 females, 3 males), with ages ranging from 18 to 53 years (M = 28.8, S.D = 8.6) took part in the experiment. All of them received a compensation of 30€ at the end of the experiment. Participants were informed that they were free to leave the experiment at any time, and that their data would be treated anonymously. The investigation was carried out in accordance with the latest version of the Declaration of Helsinki. Written informed consent was obtained from all participants involved in the study which was reviewed and approved by the local ethics committee, CPP Ile de France VII (registration number: 2022-A01597-36). All the data were anonymized before analysis.

### Stimuli

Four types of stimuli were presented to the participants:

- Monaural beats (C0 condition) obtained by adding two pure tones of different frequencies. The resulting signal is defined in Eq (2). The signal is presented simultaneously to both ears. The values of pure tone frequencies were 440 Hz and 446 Hz for the G6 group, leading to a modulating frequency of 6 Hz. For the G40 group, the frequencies were 440 Hz and 480 Hz, leading to a modulating frequency at 40 Hz. This was considered as control condition since the stimuli did not contain any spatial attributes.
- Binaural beats (C1 condition): two pure tones of different frequencies were presented separately in each ear. In the G6 group, a 440 Hz tone was presented in the left ear and a 446 Hz tone in the right ear. In the G40 group, a 440 Hz tone was presented in the left ear and a 480 Hz tone in the right ear.
- Panning beats (C2 condition): to generate the panning, a pure tone of frequency equal to the average of the two frequencies used in C1 condition (i.e., 443 Hz for the G6 group and 460 Hz for the G40 group) is modulated in amplitude by a panning law which is a sine function in the left ear, and a cosine function in the right ear [34, 35] which frequencies are 6 Hz for the G6 group and 40 Hz for the G40 group.
- Alternate beeps (C3 condition): a sequence of beeps is presented alternately in each ear at a frequency rate of 6 Hz in the G6 group, and 40 Hz in the G40 group. A single beep is constructed by modulating a sinusoid of frequency equal to the average of the two frequencies used in C1 condition (i.e., 443 Hz for the G6 group and 460 Hz for the G40 group) by a single period of a sinusoidal envelope of 80 Hz corresponding to twice the highest frequency rate used for the study, i.e. 40 Hz.

### EEG measures

The EEG recording was done using a prototype OpenBCI headset (OpenBCI, New-York, USA) made by the company Conscious Labs (Conscious Labs SAS, Paris, France). The EEG acquisition system consisted of 16 dry and active electrodes placed in accordance with the international 10/20 system [36] as follows: 2 in prefrontal (Fp1 and Fp2), 3 in frontal (F3, Fz, F4), 3 in central (C3, Cz, C4), 3 in parietal (P3, Pz, P4), 3 in occipital (O1, Oz, O2), 2 in temporal (T7 and T8) and 2 in earlobe (A1 and A2) for ground and reference. The data were acquired via the OpenBCI GUI software at a sampling frequency of 125 Hz.

### Questionnaires and task

#### Global relaxation questionnaire

We considered Dovero’s Global Relaxation Questionnaire [37], which is the French translation of The Relaxation Inventory [38]. It is a self-evaluation questionnaire that measures an individual’s subjective level of relaxation. It includes 45 statements divided into 3 scales that measure 3 dimensions of relaxation: physiological tension (PT) corresponding to physiological activities present in states of tension (15 items, maximum score = 60), cognitive tension (CT) corresponding to states of worry and anxiety (10 items, maximum score = 40) and physical assessment (PA) corresponding to a positive assessment of the general physical state (20 items, maximum score = 80). The questionnaire was presented on a computer screen using OpenSesame software [39]. Participants rated the extent to which each of the statements applied to their current state on a Likert’s scale from 0 to 4 (with 0 = not at all and 4 = very much). In the end, 3 scores were calculated, one for each scale. High scores on this questionnaire reflected a high level of relaxation.

#### Affect grid

The affect grid is a 9 x 9 Pleasure x Arousal Grid [40], which provides a global picture of the participant’s affective state at a given time with a single item. It is presented in the form of a 9 x 9 grid and includes 2 dimensions of affect, the hedonic dimension on the horizontal axis and the arousal dimension on the vertical axis. The participants were asked to indicate how they felt at that moment by placing a cross in the corresponding box on the grid presented on a computer screen using OpenSesame software [39]. The scores are given by the position of the cross along each dimension of the chosen box: rate from 1 to 9 from the left for the hedonic scale; rate from 1 to 9 from the bottom for the arousal scale.

#### Attention task

The participants performed a Not-X-Continuous Performance Task [41] using the PEBL 2.1 software [42] to evaluate their sustained attention. 360 letters were presented on the computer screen, one after the other. Each letter was presented for 250 ms. The task was divided in 6 consecutive time blocks that contained 3 sub-blocks of 20 trials. These 3 sub-blocks corresponded to 3 different inter-stimulus interval (ISI) values: 1000 ms, 2000 ms and 4000 ms. The ISI corresponds to the time between the end of the presentation of a stimulus and the beginning of the presentation of the next stimulus. In total, there were 18 ISI sub-blocks. The ISI sub-blocks were randomized so that each of the 3 ISI conditions appeared every 3 blocks but in a different order.

The instruction given to the participants was to respond as quickly as possible, by pressing the spacebar to all letters except the letter "X". The rate of appearance of the letter "X" was 10%. The task took 14 minutes. Reaction times (RTs), number of correct responses, errors of commission (when the participants pressed the spacebar to "X"), errors of omission (forgetting to respond when the presented stimulus is not an "X") and variability of RTs within participants were measured. RT variability corresponds to the variability of the standard error of RTs for each of the 18 ISI sub-blocks. When sustained attention falls, RT variability increases. Furthermore, errors of omission are associated with inattention, whereas errors of commission are more likely associated to an impulsive character of the participant [43].

### Procedure

After explaining the study and obtaining their consent, the participants were taken to an audiometric room. Participants were then assigned using an alternation method to one of the two experimental groups, noted G6 (for 6 Hz frequency beating) and G40 (for 40 Hz frequency beating). They were kept blind to group allocation. In total, each group was composed of 19 participants (G6 group: 11 males, 8 females; G40 group: 10 males, 9 females).In each group, each participant carried out 4 experimental sessions in a random order corresponding to the 4 conditions (C0, C1, C2 and C3).

**Fig 1:**
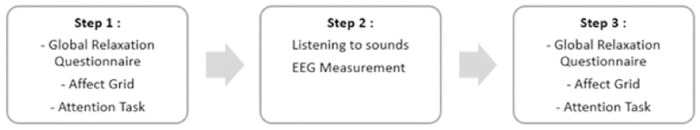
The 3 steps of the first experimental session.

**Fig 2:**
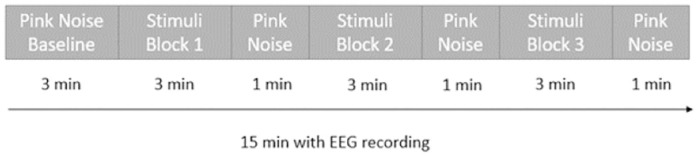
The different blocks that compose the auditory stimulation in step 2.

The first experimental session was composed of 3 steps (Fig 1). In step 1, participants started by filling in the global relaxation questionnaire and the affect grid and then, they completed the attention task. This step allowed collecting reference data about the initial cognitive state of the participants at the beginning of the experiment. After that, the EEG headset was placed. The participants were asked to put on in-ear headphones (Sennheiser IE100 Pro). Once equipped, in step 2, sounds corresponding to one of the 4 stimuli conditions were presented. The participants were instructed to sit comfortably, relax and listen carefully to the sounds. This step lasted for 15 min and was divided into 4 blocks: a first block of 3 minutes of pink noise allowing the recording of physiological data at rest (considered as baseline), then 3 successive blocks of 3 minutes of stimuli. Each block was followed by 1 minute of pink noise (Fig 2). Once the auditory stimulation was over, in step 3, the EEG equipment was removed, and the participants completed the questionnaire, the affect grid and the attention task again.

In the three remaining experimental sessions, corresponding to the remaining stimuli conditions, steps 2 and 3 were reiterated. Overall, each participant experienced all four types of stimuli in four distinct experimental sessions. The order of stimuli conditions was randomized across participants. To avoid excessive fatigue of the participants, the experiment took place over 2 days separated by one week. The participants did 2 experimental sessions in a day. The total duration of the experiment was about 3h40, including 2h for the first day and 1h40 for the second day.

### Data analyses

#### EEG data

The EEG data were processed in MATLAB with the EEGLAB toolbox [44]. First, the data were filtered with a 0.4 Hz high-pass filter and a notch at 50 Hz. As a precaution, we used a notch filter centered at 25 Hz on all data to avoid sub-harmonic components linked to the 50 Hz power supply. Then, the faulty electrodes were removed. Given the small number of sensors, missing signals were interpolated if only one electrode was removed (i.e., if no more than 10% of the signal was missing). After that, the signal was inspected and cleaned by visual inspection to remove large artifacts. Then, 10-second epochs were extracted from the continuous signal. Finally, an Independent Component Analysis (ICA) was conducted on the epochs and the component corresponding to eye movements was removed.

After the preprocessing phase, frequency analyses were conducted on the EEG data. The broadband EEG power spectrum was divided into five standard frequency bands: Delta (1–4 Hz), Theta (5–8 Hz), Alpha (9–12 Hz), Beta (13–30 Hz), and Gamma (32–44 Hz). Absolute and relative power was calculated for each frequency band. Only the relative power was statistically analyzed. The power calculation was done for each epoch and for each electrode. Then, the 10-second epochs were averaged for each stimulation block (i.e., the baseline and the 3 stimulation blocks, see Procedure section). Finally, the electrodes were grouped, and the relative power was averaged by the 5 following regions of interest (ROI): frontal (F3, Fz, F4), central (C3, Cz, C4), parietal (P3, Pz, P4), occipital (O1, Oz, O2) and temporal (T7, T8). The spectral power of the baseline was subtracted from the spectral power of each stimulation block. This was done separately for each frequency band, each electrode and at the participant level. Then two-factor repeated measures ANOVAs were performed for each group (G6 and G40) with stimulus condition (C0, C1, C2 and C3) and block (Block 1, 2 and 3) as factors, followed by post-hoc Tukey tests. Statistical significance was defined as p<0.05. Statistical analyses were conducted with RStudio software ([45], v2022.07.02; R core v4.2.3; Tidyverse v2.0.0; Rstatix v0.7.2).

#### Behavioral data

For the PT, CT and PA scores measured with the Global Relaxation Questionnaire as well as the scores obtained from the affect grid (arousal and hedonism), one factor repeated measures ANOVAs were performed for each group (G6 and G40) with stimulus condition (C0, C1, C2 and C3) to which was added the pre-experimental phase (Step 1 of the first session) as a factor, provided that the hypotheses of normality of the residuals and homogeneity of the variances were respected. If this was not the case, then non-parametric Friedman tests were conducted. For post-hoc tests, a Tukey test was performed after an ANOVA, and a pairwise Wilcoxon Signed-Rank test comparisons, with p-value corrected into q-value, using Benjamini-Hochberg method for false discovery rate corrections, after a Friedman test. The same analysis was performed with data collected from the attention task, i.e., on the errors of omission, the errors of commission and reaction times (RTs) for correct responses. For RT variability, a two-factor repeated measures ANOVA was performed for each group with stimulus condition (C0, C1, C2 and C3) and block (Block 1, 2 and 3) as factors. Statistical significance was defined as p < 0.05. Statistical analyses were conducted with RStudio software ([45], v2022.07.02; R core v4.2.3; Tidyverse v2.0.0; Rstatix v0.7.2).

## Results

### Results for the binaural beats (C1) condition

Regarding the EEG results comparing monaural (C0) and binaural beats (C1) conditions, the G40 group did not exhibit any differences in relative spectral power across frequency bands. However, in the G6 group, specifically in the parietal region, Alpha power was significantly lower in condition C1 than in C0 [*F*(3,27) = 4.515, *p* < 0.05, ${ŋ}_{f}^{2}$ = 0.149].

Turning to the behavioral outcomes, in the G40 group, a significant stimulus effect was observed on the CT scale [χ^2^(4) = 19.5, p < 0.001, W = 0.256], with higher scores for condition C1 (score = 33.789, *q* < 0.01, *p* < 0.001) compared to the scores in the pre-experimental phase. Additionally, a significant effect was found on the arousal dimension [χ^2^(4) = 13.0, p < 0.05, W = 0.171], with lower scores for condition C1 (score = 3.316, *q* < 0.05, *p* < 0.01) compared to the scores in the pre-experimental phase. A similar tendency was observed for the PA scale [*F*(2.72,48.97) = 3.827, *p* < 0.05, ${ŋ}_{f}^{2}$ = 0.066], although this result did not survive a post-hoc Tukey test. In the G6 group, a significant effect of the stimulus on the CT scale was also found [χ^2^(4) = 1928, *p* < 0.01, W = 0.252], with higher scores for condition C1 (score = 32.947, *q* < 0.05, *p* < 0.01) compared to the scores in the pre-experimental phase. None of these effects were observed in the C0 condition. Regarding the sustained attention task, no significant results were identified.

### Results for the panning beat (C2) and alternate beeps (C3) conditions

**Fig 3:**
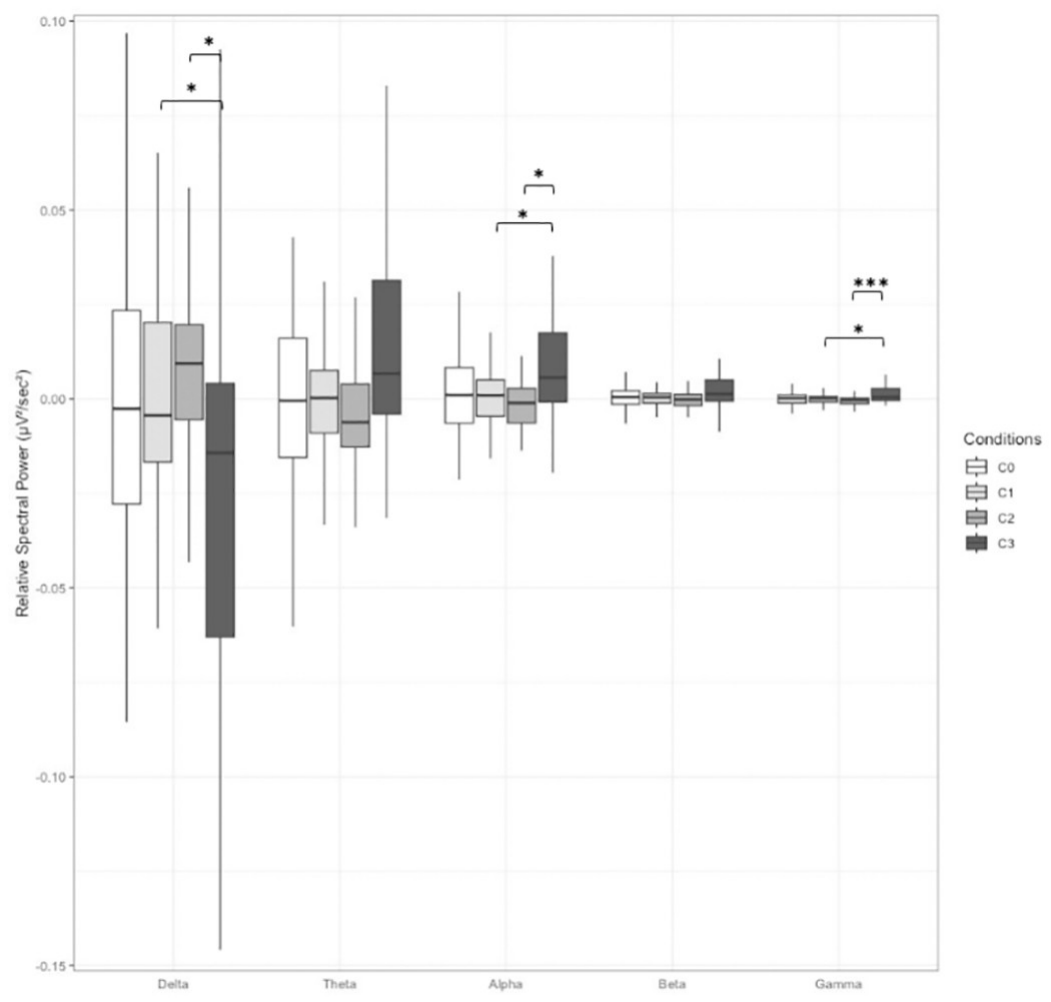
Relative spectral power for the G40 group, averaged over all electrodes, for all frequency bands and for all stimulus conditions. *C0*: Monaural Beat; *C1*: Binaural Beat; *C2*: Panning Beat; *C3*: Alternate Beep. Error bar indicates s.d. *: *p* < 0.05; **: *p* < 0.01, ***: *p* < 0.001.

In the G40 group, the stimulus had a significant impact on the relative spectral power averaged over all electrodes across the Alpha [*F*(2.10,27.34) = 5.057, *p* < 0.05, ${ŋ}_{f}^{2}$ = 0.125], Delta [*F*(1.97,25.59) = 4.165, *p* < 0.05, ${ŋ}_{f}^{2}$ = 0.138], and Gamma [*F*(3,39) = 5.391, *p* < 0.01, ${ŋ}_{f}^{2}$ = 0.167] frequency bands. Post-hoc tests revealed that relative spectral power was higher in condition C3 compared to C1 and C2 in the Alpha and Gamma bands, while the opposite tendency was observed in the Delta band (Fig 3). No significant effects were found by region of interest (ROI).

**Fig 4:**
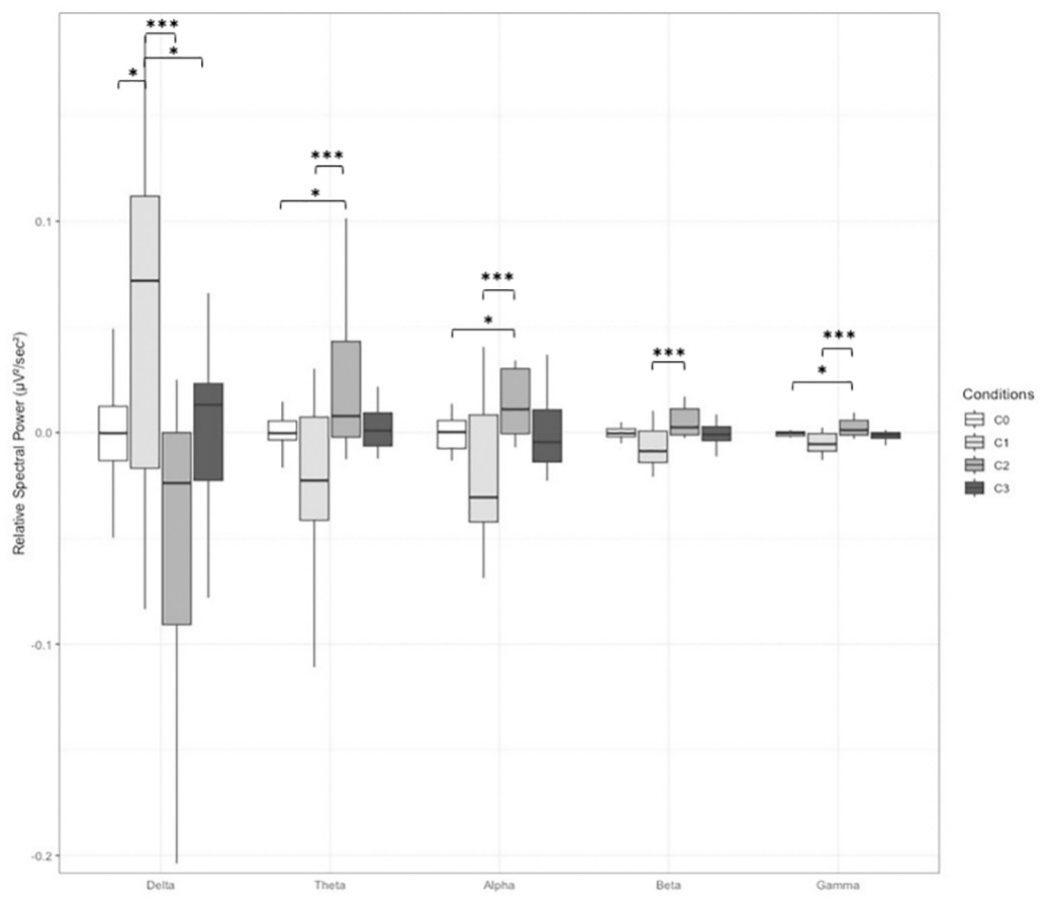
Relative spectral power for the G6 group, averaged over the temporal electrodes (T7 and T8), for all frequency bands and for all stimulus conditions. *C0*: Monaural Beat; *C1*: Binaural Beat; *C2*: Panning Beat; *C3*: Alternate Beep. Error bar indicates s.d. *: *p* < 0.05; **: *p* < 0.01, ***: *p* < 0.001.

In the G6 group, the stimulus had a significant effect on the relative spectral power averaged over all electrodes in the Beta [*F*(3,36) = 3.916, *p* < 0.05, ${ŋ}_{f}^{2}$ = 0.117] and Gamma frequency ranges [*F*(3,36) = 3.069, *p* < 0.05, ${ŋ}_{f}^{2}$ = 0.086]. Specifically, in the Gamma band, power was higher in condition C2 than in C1 (*p* < 0.01) and C3 (*p* < 0.05), and in the Beta band, it was higher in condition C2 than all other conditions (C2 vs. C0: *p* < 0.05; C2 vs. C1: *p* < 0.001; C2 vs. C3: *p* < 0.05). In the parietal region, Alpha power was higher in C2 than in C1 (*p* < 0.001) and C3 (*p* < 0.01). Furthermore, in the Delta band, power was lower in C2 than in C1 (*p* < 0.01) and C3 (*p* < 0.01). In the temporal region, significant stimulus effects were observed across all frequency bands [Alpha: *F*(3, 15) = 4.233, *p* < 0.05, ${ŋ}_{f}^{2}$ = 0.251, Beta: *F*(3,15) = 4.599, *p* < 0.05, ${ŋ}_{f}^{2}$ = 0.268, Theta: *F*(3,15) = 6.036, *p* < 0.01, ${ŋ}_{f}^{2}$ = 0.299, Delta: *F*(3,15) = 6.335, *p* < 0.01, ${ŋ}_{f}^{2}$ = 0.309, Gamma: *F*(3,15) = 4.839, *p* < 0.05, ${ŋ}_{f}^{2}$ = 0.268]. In the Alpha, Gamma, and Theta bands, the spectral power was higher in condition C2 than in C1 and C0 (Fig 4). The spectral power was also higher in C2 than in C1 in the Beta band. Finally, the relative power in the Delta band was lower in C2 than in C0 and C1 and higher in C1 than in C3.

**Fig 5:**
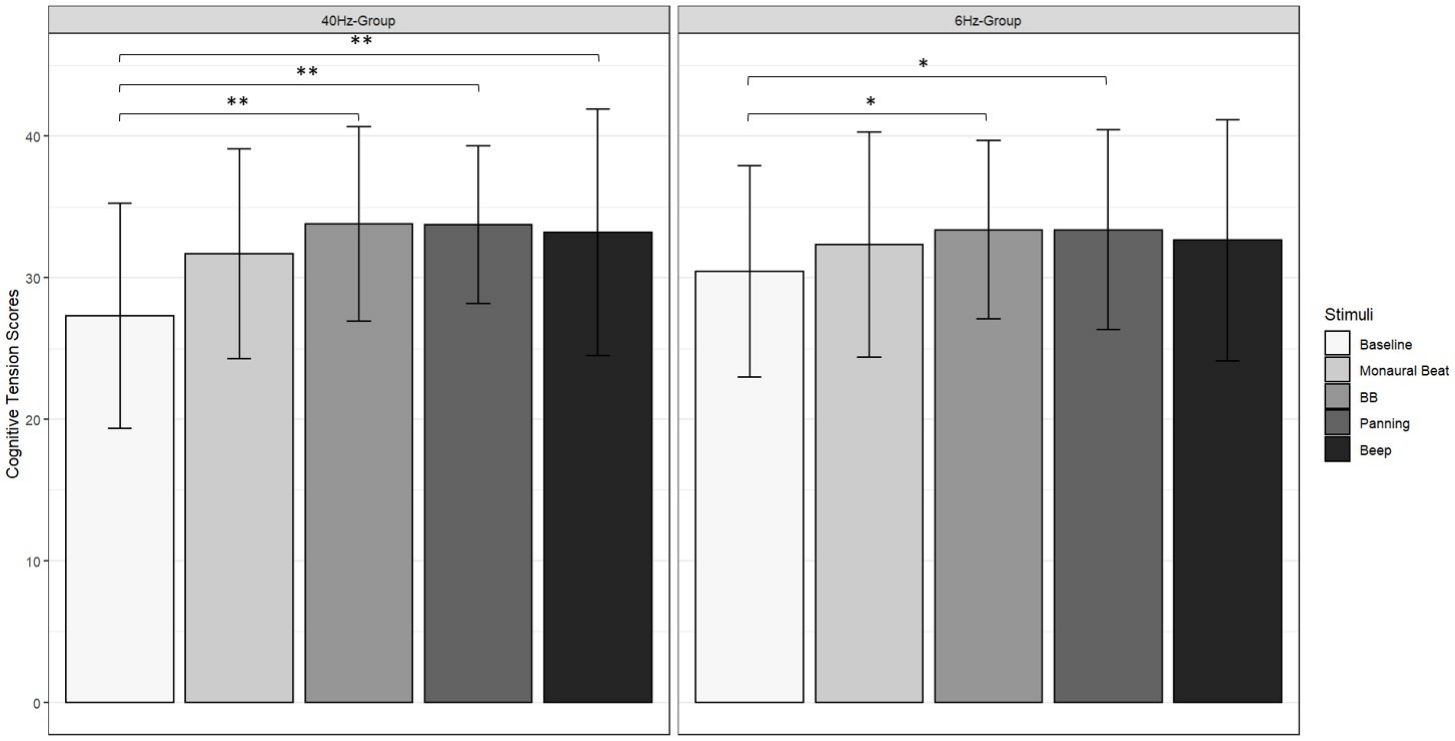
Participants’ scores on the cognitive tension (CT) scale of the relaxation questionnaire. *Left*: G40 group; *right*: *G*6 group. *Baseline*: Pre-experimental score; *C0*: Monaural Beat; *C1*: Binaural Beat; *C2*: Panning; *C3*: Beep. Error bar indicates s.d. *: *q* < 0.05; **: *q* < 0.01.

**Fig 6:**
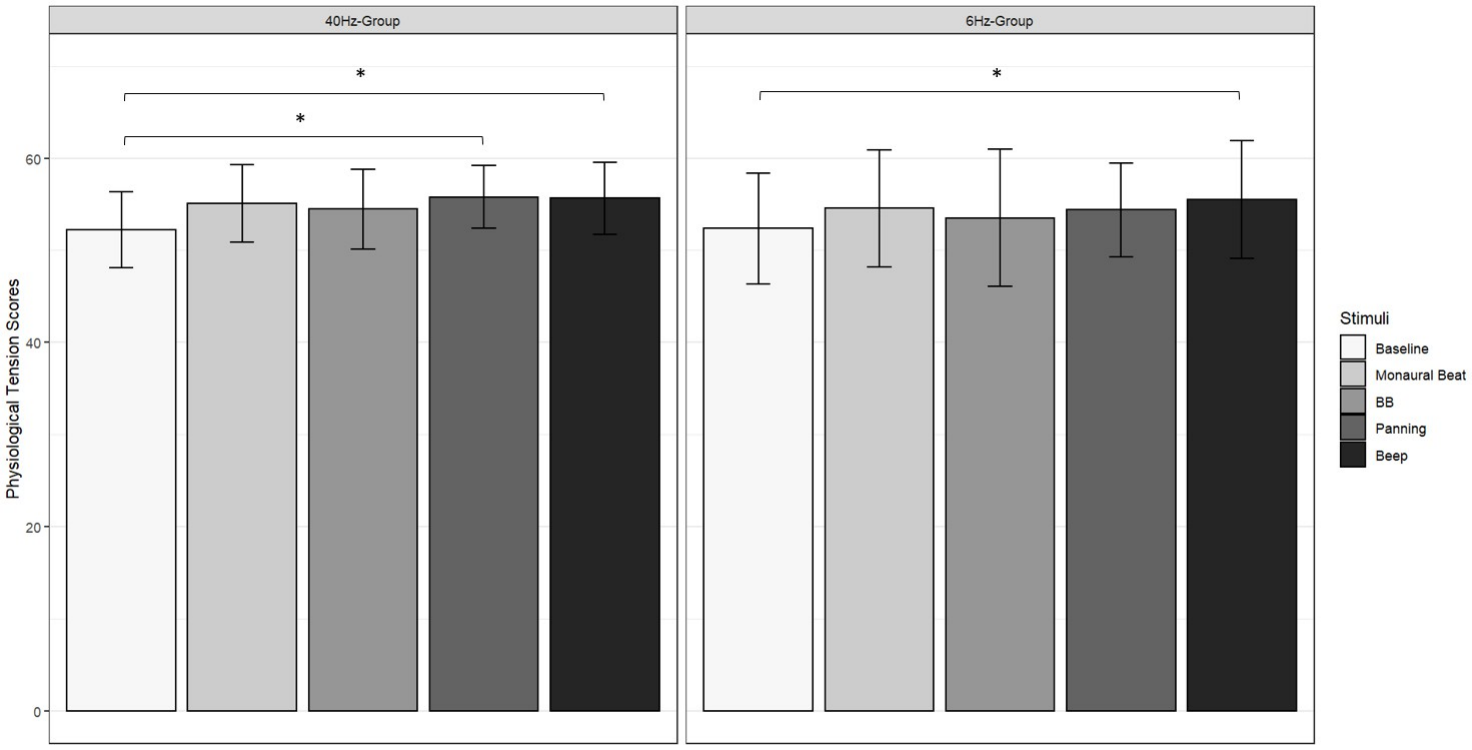
Participants’ scores on the physiological tension (PT) scale of the relaxation questionnaire. *Left*: G40 group; *right*: *G*6 group. *Baseline*: Pre-experimental score; *C0*: Monaural Beat; *C1*: Binaural Beat; *C2*: Panning; *C3*: Beep. Error bar indicates s.d. *: *q* < 0.05.

Moving to behavioral data, in the G40 group, a significant stimulus effect was found on the CT scale [χ ^2^(4) = 19.5, *p* < 0.001, W = 0.256] with higher scores for conditions C1 (score = 33.789, *q* < 0.01, *p* < 0.001), C2 (score = 33.737, *q* < 0.01, *p* < 0.01) and C3 (score = 33.211, *q* < 0.01, *p* < 0.01) compared to the pre-experimental score (score = 27.316, Fig 5, left). On the PT scale a significant effect was also observed [χ ^2^(4) = 10.4, *p* < 0.05, W = 0.137] with higher scores for conditions C2 (score = 55.789, *q* < 0.05, *p* < 0.01) and C3 (score = 55.632, *q* < 0.05, *p* < 0.01) compared to the pre-experimental score (score = 52.211, Fig 6, left). A similar effect was observed on the arousal dimension [χ ^2^(4) = 13.0, *p* < 0.05, W = 0.171] with lower scores in conditions C1 (score = 3.316, *q* < 0.05, *p* < 0.01) and C2 (score = 3.053, *q* < 0.05, *p* < 0.01) compared to the pre-experimental score (score = 4.789). A similar trend was noted for the PA scale [*F*(2.72,48.97) = 3.827, *p* < 0.05], however this result failed to survive a post-hoc Tukey test.

In the G6 group, a significant stimulus effect was observed on the CT scale [χ ^2^(4) = 19.2, *p* < 0.01, W = 0.252] with higher scores in conditions C1 (score = 32.947, *q* < 0.05, *p* < 0.01) and C2 (score = 33.158, *q* < 0.05, *p* < 0.01) compared to the pre-experimental score (score = 29.789, Fig 5, right). The same trend was observed on the PT scale [χ^2^(4) = 12.1, *p* < 0.05, W = 0.159] with an increase in score in condition C3 (score = 55.789, *q* < 0.05, *p* < 0.01) compared to the pre-experimental score (score = 53.053, Fig 6, right). Regarding the attention task, no significant differences were found between stimulus conditions in either group, for reaction times (G40 group: *p* = 0.911; G6 group: *p* = 0.237), commission errors (G40 group: *p* = 0.884; G6 group: *p* = 0.41) nor omission errors (G40 group: *p* = 0.833; G6 group: *p* = 0.463). There was a significant decrease in the variability of the RT as a function of the blocks in both groups [G40 group: *F*(17,306) = 3.566, *p* < 0.01; G6 group: *F*(17,255) = 2.067, *p* < 0.01], but the interaction between the condition and the block was not significant (G40 group: *p* = 0.604; G6 group: *p* = 0.519).

## Discussion

The aims of this study were to determine if specific auditory modulations could elicit modifications in brain activity, and in particular, if these effects can be induced by spatial attributes of the sounds. Several hypotheses were formulated from the literature. The first one was that BBs would lead to an increase of spectral power in the corresponding frequency bands, and an increase in relaxation with 6Hz stimulation and an improvement in sustained attention with 40 Hz stimulation compared to the control condition. The second hypothesis was that these effects are due to the presence of spatial attributes contained in the sounds. Therefore, these effects would be even more important with alternating beeps, panning beats than with BBs.

Our results in EEG with 6 Hz BBs showed an increase in the power within the Theta frequency, but showed a decrease in the power within the Alpha frequency band compared with the monaural beat condition in the parietal region. Conversely, at 40 Hz, our findings indicated a lack of significant effect on EEG attributed to BBs. In practice, the 40 Hz frequency was chosen in our study due to its prevalence in literature concerning BB’s impact on attention [24, 25, 46]. At this frequency, we also approach the upper limit of the perceptual threshold of BB, where the two sine waves are more likely to be perceived separately. It is noteworthy that the proximity of such BB disappearance threshold poses a notable constraint in the studies on BB phenomenon, potentially contributing to the absence of consensus and the variability of results observed in the literature. Concerning the relaxation and affect questionnaires, we found that BBs improved cognitive relaxation at both 6 Hz and 40 Hz. However, a decrease in arousal was observed only with 40 Hz BBs. Our results did not show any significant effect of BBs on the performance in the sustained attention task. Taken together, these results suggest that opposed to the often-emphasized hypothesis that the effect of BBs on cognition and emotional states is induced by an increase in spectral power in the frequency band corresponding to that of the BB, the modification of brain activity is not a prerequisite for an improvement in relaxation. Similarly, the idea that an increase in relaxation would only be possible with low-frequency BB is not supported by our findings. Furthermore, these results with 6 Hz BBs are in line with those of [20, 21] who observed a decrease in anxiety scores in participants listening to BB. Furthermore, Gantt et al. [22] found a reduction in stress with the use of BB, yet our results showed an improvement in cognitive relaxation and not in physiological relaxation. This may be explained by the type of measurements used. Indeed, Gantt et al. [22] measured heart rate variability, whereas our measures was based on participants’ subjective relaxation evaluated by a questionnaire. Thus, future studies could couple electrophysiological and self-reported measures of relaxation for a better understanding of the phenomenon.

Furthermore, we found that all tested stimuli, except the monaural beats, had a positive effect on relaxation. Specifically, at 6 Hz, both BB and panning beats improved cognitive relaxation, whereas alternate beeps enhanced physiological relaxation. The changes in EEG and relaxation observed with these auditory stimulations but not with monaural beats (which only induce a perception of beats without displacement) support the hypothesis that the effect of sound modulations is linked to spatial attributes rather than the sensation of beating itself. We also observed that the auditory stimulations led to different brain activity patterns. In particular, at 6 Hz, we identified two distinct patterns of electrical brain activity: on the one hand a decrease in very low frequency range (Delta) and an increase in all other bands observed with panning beats, and on the other hand, the opposite, i.e., an increase of the power in the Delta band and a decrease in the other frequency bands observed with BB and alternate beeps. These patterns are more pronounced in the temporal regions, where the auditory cortices are located. These results are in line with the theory of Pagani et al. [47] on the mechanism of action of EMDR (Eye Movement Desensitization and Reprocessing) and bilateral sound, and slow wave sleep. This explanation will be detailed later in the discussion in view of its clinical nature. At 40 Hz, alternating beeps resulted in an increase in Alpha and Gamma bands (generally associated with intense mental activities) and a decrease in Delta band (associated with deep sleep) compared with the BB and panning beat conditions. Thus, these brain activity patterns confirm that BBs can be likened to a source displacement, but they also appear to be influenced by the stimulation frequency. Note that a limitation of these results is the large variability across participants (indicated by the error bars in Figs 3 and 4) around the mean spectral power especially in the Delta band and, to a lesser extent, the Theta band. Future research could explore a broader frequency range to gain a more comprehensive understanding of this phenomenon.

Finally, the similar relaxation outcomes between BBs and panning beats at 40z, despite different EEG patterns, suggest that the nature of the stimulation itself plays a more important role in relaxation than the modifications of cerebral activity that it causes. This result will have to be confirmed by using more precise electrophysiological measurement equipment such as EEG headset with a larger number of electrodes or magnetoencephalogram device (MEG). At 40 Hz, effects on participants’ subjective relaxation were observed. In particular, BBs, panning beats and alternate beeps improved cognitive relaxation while physiological relaxation was increased only with panning beats and alternate beeps. Furthermore, alternate beeps have a positive impact on relaxation while avoiding the feeling of fatigue. Note that we observed the opposite for BB and panning beats, meaning that such stimuli rather induced a feeling of sleepiness to the participants. We hypothesized that the effects observed would be greater for stimuli evoking perceptible moving sound sources than with BBs for which movement is not perceptible at high frequency. Taken together, our results showed a different effect rather than a larger effect of these stimuli on EEG and relaxation. It would seem, therefore, that it is the type of spatially moving sounds rather than the perceptibility of motion from a spatialised sound source that has an impact on the observed phenomenon.

Our results did not show any significant effect of the auditory stimulation on sustained attention. This lack of result can be explained by the task used. In the literature, positive results concerning the improvement of attention by BB within the Gamma frequency range have been reported [24, 25, 46]. However, the tasks used most often measure focused attention or cognitive control. Some authors have hypothesized that BB allow for better resource distribution [24, 25]. One study investigated sustained attention with BB but did not observe any effect [26]. Analyses of RT variability showed a main effect of the block, which indicates a fluctuation of sustained attention during the task. However, this variation did not depend on the stimulus, which means that attention fluctuated over the course of the task, but the stimuli tested had no impact on this fluctuation. It would therefore be interesting, in future studies, to investigate the effect of such stimuli on distributed or focused attention.

Finally, our results are relevant in the context of the Eye Movement Desensitization and Reprocessing (EMDR) therapy, an efficient method for post-traumatic stress disorder, although the brain mechanisms underlying the effect are still unknown. The EMDR therapy was discovered by Dr. Francine Shapiro in 1987. This therapy combines recall of traumatic memory and bilateral stimulation initially practiced using eye movements [48]. Recent findings [49, 50] showed a major effect on cognitive states in people, directly related to the discontinuous nature of bilateral visual stimuli, compared to the continuous character of intermittent stimulations or non-intermittent bilateral stimulation. These same authors stated that bilateral alternating stimulation (BAS) also works with auditory or somesthetic (tactile) stimulations [51, 52]. In one study, Boukezzi et al. [53] used auditory BAS of 1 Hz frequency in a fear conditioning protocol. Stress responses were measured via electrodermal activity. Results showed that during the recall phase of extinction, the amplitude of Skin Conductance Responses (SCRs) was lower when participants received bilateral stimuli than when they did not. On the other hand, there was no difference with respect to expectation, i.e., all participants equally expected to receive electric shocks only, those who had bilateral stimulations were less stressed than the others. Although the context of our experiment differed, our results point in the same direction. The alternate beeps at 6 Hz and 40 Hz appear to be effective in reducing stress, even if we observed that participants’ pre-experimental scores on the relaxation questionnaire were already high. To get larger differences in these scores during the experiment, it might therefore be relevant for future research to choose a questionnaire that is more sensitive to variations in relaxation in healthy individuals. Another alternative would be to induce stress in participants at the beginning of the experiment, such as a fear conditioning protocol as done by [53]. Finally, it would also be interesting to test these stimuli on a population with anxiety disorders to see if the stimuli provide any benefit to them.

There is currently no agreement on the mechanisms responsible for the effectiveness of EMDR. However, several theories explaining the effectiveness of EMDR have been proposed. One theory, developed by Pagani [47], suggests that EMDR mimics the physiological processes during the deep slow-wave sleep phase. This phase is characterized by an increase in Delta waves. Several studies [54, 55] have supported this theory by demonstrating that alternating stimulations produce slow waves similar to those observed during deep slow-wave sleep. This in accordance with the increase of Delta power in temporal region we observed with 6 Hz BBs and alternating beeps. Nevertheless, this impact of BAS on Delta waves is observed in frontal area rather than temporal area. It is worth noting that these studies used visual BAS, not auditory ones. Another hypothesis underlying the EMDR therapy is based on the alternating nature of the stimulations, which causes desensitization, regardless of the sensory stimulations (visual, tactile or auditory, [50]). However, to our knowledge, there is still no study aiming at questioning the specific nature of the stimuli (visual, auditory or tactile) with regard to the effects obtained in the practice of EMDR. This work would therefore benefit from being carried out so that the consensus hypothesis can be tested, for instance, by protocols based on the results obtained in alternate beeps condition.

## Conclusion

In summary, our study investigated the impact of auditory modulations, particularly induced by spatial attributes within sounds, on brain activity and subjective states. Our results showed that the effect of binaural beats on human was related to the spatial characteristics of the sound. Improvement of relaxation was associated with a modification of brain electrical activity with spatially moving sounds compared to auditory stimulation that does not induce a perception of movement (monaural beats). However, there was no notable effect on performance in the attention task (errors and reaction times). At 6 Hz, two patterns of electrical activity were observed: with panning beats, there was a decrease in Delta activity and an increase in all other frequency bands, while with binaural beats and beeps, the opposite pattern emerged, with an increase in Delta activity and a decrease in all other frequency bands. At 40 Hz, there was an increase in Gamma and Alpha activity and a decrease in Delta activity with alternating beeps. Overall, our findings contribute to a deeper understanding of how auditory modulations influence cognition and emotion, suggesting potential therapeutic applications in clinical and more generally care contexts.

## Supporting information

S1 Checklist Reporting checklist for randomised trial. (DOCX)
